# Horizontal Eye Position Affects Measured Vertical VOR Gain on the Video Head Impulse Test

**DOI:** 10.3389/fneur.2015.00058

**Published:** 2015-03-17

**Authors:** Leigh A. McGarvie, Marta Martinez-Lopez, Ann M. Burgess, Hamish G. MacDougall, Ian S. Curthoys

**Affiliations:** ^1^Department of Neurology, Institute of Clinical Neurosciences, Royal Prince Alfred Hospital, Camperdown, NSW, Australia; ^2^Department of Otorhinolaryngology, Clinica Universidad de Navarra, Pamplona, Spain; ^3^Vestibular Research Laboratory, School of Psychology, University of Sydney, Sydney, NSW, Australia

**Keywords:** vestibular, vestibulo-ocular reflex, vHIT, semicircular canal, eye movement

## Abstract

**Background/hypothesis:** With the video head impulse test (vHIT), the vertical VOR gain is defined as (vertical eye velocity/vertical head velocity), but compensatory eye movements to vertical canal stimulation usually have a torsional component. To minimize the contribution of torsion to the eye movement measurement, the horizontal gaze direction should be directed 40° from straight ahead so it is in the plane of the stimulated canal plane pair. *Hypothesis*: as gaze is systematically moved horizontally away from canal plane alignment, the measured vertical VOR gain should decrease.

**Study design:** Ten healthy subjects, with vHIT measuring vertical eye movement to head impulses in the plane of the left anterior-right posterior (LARP) canal plane, with gaze at one of five horizontal gaze positions [40°(aligned with the LARP plane), 20°, 0°, −20°, −40°].

**Methods:** Every head impulse was in the LARP plane. The compensatory eye movement was measured by the vHIT prototype system. The one operator delivered every impulse.

**Results:** The canal stimulus remained identical across trials, but the measured vertical VOR gain decreased as horizontal gaze angle was shifted away from alignment with the LARP canal plane.

**Conclusion:** In measuring vertical VOR gain with vHIT the horizontal gaze angle should be aligned with the canal plane under test.

## Introduction

The new video head impulse test (vHIT) provides objective, accurate measures of the semicircular canal response to its physiological stimulus – the angular acceleration during head rotation (1–4). The subject is instructed to maintain fixation on an earth-fixed target at a distance of about a meter while the operator delivers small, brief, passive, head turns in the plane of the semicircular canals being tested. These turns (head impulses) are unpredictable in size, direction, velocity, and timing, and are measured exactly. The compensatory eye movement response is recorded by a small, light, fast, head-mounted camera to measure the VOR. This is a major step forward in clinical evaluation of peripheral vestibular function.

**Figure 1:**
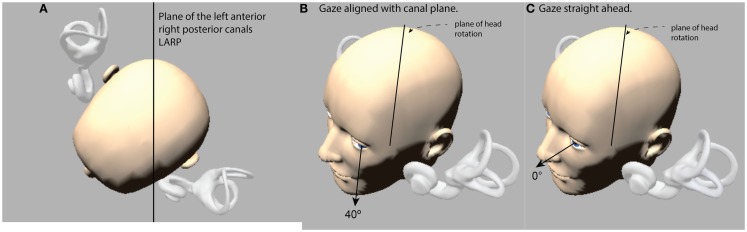
**(A)** View looking down on a schematic head with enlarged canals to show the approximated planes of the vertical canals. **(B)** The optimal horizontal eye position for using vHIT to measure vertical canal function: it is with gaze aligned with the canal plane under test (this is labeled 40° in the figures below). **(C)** If gaze is straight ahead (0°), the prediction is that the vertical component becomes much smaller. (The images are stills from the free iPhone app aVOR at the app store).

However, there is an issue in applying vHIT to measure vertical semicircular canal function not encountered in testing the horizontal canals. The vertical canals are oriented in planes about 45° to the median plane of the head (see Figure 1A) and form two matched pairs – left anterior-right posterior (LARP) and right anterior-left posterior (RALP) (5). To test canal function, the head impulses must be delivered in the plane of the canal pair under test: so simply pitching the head forward or backward in a sagittal plane stimulates all vertical canals and does not allow for identification of which particular vertical canal may be dysfunctional (6, 7). However, since the LARP plane, the RALP plane, and the plane of the lateral semicircular canals are roughly orthogonal to each other (5), a head impulse in the plane of one pair of canals will stimulate mainly that pair, and not the other two semicircular canal pairs. In this way, a deficit in one vertical canal is identified by the reduced response for the impulse, which activates that particular canal. This has been shown with search coils (7, 8), where loss of a single vertical canal was identified. However, in measuring vertical canal function with the present algorithm of vHIT, which does not measure torsion, there is an additional major issue – the horizontal gaze direction during the vertical canal plane stimulation.

Measures of the eye movement response by scleral search coils in response to LARP and RALP impulses show that with gaze in the primary position the compensatory eye movements for head pitches in the LARP or RALP planes are a combination of vertical and torsional components (9, 10). In principle what determines the relative contributions of the vertical and torsional components is the horizontal position of the eye in the orbit. But for reasons of speed, simplicity, and accuracy, the present version of vHIT (Otometrics impulse) is a two-dimensional recording system – so it only measures the horizontal and vertical eye movement components. For vHIT to be able to record vertical VOR to vertical head impulses, it is necessary to arrange the test parameters so the torsional component of the response is minimal. The search coil studies showed that this can be achieved if during a LARP plane stimulus the horizontal eye position is far lateral – with gaze directed along a line parallel to the LARP plane of the vertical canal pair under test (Figure 1B) (10). With that far lateral horizontal gaze direction the compensatory eye movement is almost purely vertical with negligible torsion.

In the vHIT technique, vertical VOR gain is defined as the ratio of the vertical eye movement response to the vertical head movement stimulus, so the prediction is that for any one subject, the measured vertical VOR gain should decrease as gaze position deviates horizontally away from canal plane alignment. So in using vHIT for exactly the same vertical head movement stimulus, changing only the horizontal eye position is expected to change the vertical eye movement response (see Figure 1C), so the prediction is that measured vHIT vertical VOR gain will decrease as horizontal eye position shifts away from canal plane alignment. We tested that prediction on 10 healthy asymptomatic subjects using Otometrics impulse prototype system in a well lit room (to ensure a small pupil).

If the head pitch stimulus remained exactly the same for all head impulses, but the direction of gaze in the horizontal plane moved away from being aligned with the canal pair under test, we predicted the vertical eye movement component should correspondingly decrease.

## Materials and Methods

### Subjects

All test subjects were healthy, active, community dwelling individuals with no record of vestibular or central pathologies. They gave informed consent. The procedures followed were in accordance with the ethical standards of the Helsinki Declaration and were approved by the Sydney Local Health District Ethics Review Committee (Royal Prince Alfred Hospital zone), Protocol number: X11-0085 HREC/11/RPAH/104.

### Eye movement recording

The measurement of VOR by vHIT has been described in detail previously (1–4). Subjects were seated in a height-adjustable, rotatable office chair so that their head was placed at the ideal height for the operator to deliver horizontal and vertical head impulses. The goggles were tightened on the head until movement of the goggles at the bridge of the nose was an absolute minimum as tested by a gentle lateral tug on the goggles by the operator. With some Asian subjects lacking a prominent nose bridge, a firm foam insert was wedged between the goggles and the nose, to fill the gap between the goggles and the bridge of the nose and so minimize goggle movement. Saccades to known fixed targets were used to calibrate the system, and this calibration was then checked by the tester rocking the subject’s head slowly sinusoidally back and forth in the plane of the vertical canals under test while the subject fixated the fixation target, to ensure that head velocity and eye velocity traces exactly overlaid on the display. Room lighting conditions were adjusted to ensure that the pupil was small and the pupil image was not affected by reflections onto the pupil image at any point in the range of the head movement.

The vHIT stimulus consisted of passive short, sharp head rotations, delivered in unpredictable direction, and magnitude with minimal “bounce-back” at the end of the head impulse: each head impulse was “turn and stop.” All tests were performed by the same right-handed operator. Subjects were instructed to try to maintain fixation on the target and if they lost it to try to regain it as quickly as possible.

To test the left anterior-right posterior pair of canals (LARP) the subject was turned toward their right and positioned en bloc so that the mid-sagittal plane of the subject’s body and head were aimed 40° to the right of the fixation point. During the LARP test, the body and head stayed aligned and both were aimed 40° to the right of the fixation point. The subject was instructed to fixate the central fixation point at eye level at a distance of 1.2 m. To do that the person must look out of the left corner of their orbits, and look along an earth-horizontal line, which is close to being parallel to the plane of the vertical canal pair (LARP) under test (Figure 1B). With this far lateral horizontal eye position, the compensatory eye movement for stimulation of the LARP canals is almost a purely vertical eye movement: a head pitch forward (toward the target) activates the left anterior canal and causes a vertically upward eye movement, and a head pitch back (away from the target) activates the right posterior canal and causes a vertically downward eye movement, so the vHIT vertical VOR gain should be close to 1.0.

Vertical canal head impulses were performed with the right hand on the top of the subject’s head, well away from the goggles strap, and the left hand wrapped under the subject’s chin, so that the skin on the cheeks was not pushed by the fingers or the heel of the palm, ensuring there was no camera slippage. The operator aimed to maintain peak head velocities between 150°/s and 200°/s for each gaze angle.

Vertical impulses tend to be more likely to be affected by artifacts than horizontal impulses. During the vertical impulses, the operator monitored the real-time display of the traces to check that the eye trace was moving only in the vertical plane, with no significant horizontal motion. An important artifact to avoid is the eyelid artifact, which is a particular problem in testing vertical canal function because the head movement causes the pupil to be driven toward the eyelids. If the eyelid obscures part of the pupil, the software records this as a vertical eye movement. The usual situation where this occurs is that the upper eyelid slides down and obscures the top of the pupil during the head impulse. This will cause an apparent change in eye velocity. This artifact can be prevented during the preliminary set-up by ensuring that the pupil is not obscured at all during the head movement – if needed to use a different fixation target a few centimeters directly below or directly above the original fixation target. In extreme cases, it may even be necessary to use medical tape to tape the upper eye-lid up during the test, or in some people to tape the lower eye-lid down. This is only for a short time, and it is not painful and can be vital for obtaining accurate artifact-free data.

While occurring much more often in anterior canal impulses, the eyelid artifact is more difficult to detect in posterior canal impulses, as it can appear to be a covert saccade. Operator vigilance is required to recognize these artifacts. The camera operates at 250 frames/s, but the PC screen image is only updated at the standard 50 or 60 Hz, so it is possible for this artifact to occur without being noticed on the real-time screen image, even though it will appear on the digitally recorded eye velocity data trace. Hence the operator should switch between “threshold view” and “true view” to catch any eyelid movement. If it appears, taping the upper eyelid back to the forehead will usually eliminate the problem; however, in rare cases, the eyelid flick is a strong reflex that the subject has little control over, and it is not possible to eliminate. In this case, the data must be considered unacceptable for any numerical calculations.

The 10 healthy subjects were tested with multiple head impulses (around 20 in each direction) in the planes of the vertical canals at 5 different gaze angles (40°, 20°, 0°, −20°, −40°) between gaze direction aligned with the canal (40° in the graph) through to −40°.

### Statistical analysis

In this experiment, only LARP stimuli were presented, and the data for leftward and rightward head impulses were analyzed, in a two-way ANOVA with repeated measures using SPSS Version 21 (11). The main factors were impulse direction (leftwards–rightwards) and Gaze Direction angle (40°, 20°, 0°, −20°, −40°), and each of the 10 subjects was tested in all conditions. Shapiro–Wilk tests of normality showed that the assumption of normality of distribution of the raw data was accepted in all conditions. We used a two-way repeated measure ANOVA to analyze the data, and the level of statistical significance was set at *p* < 0.05. Mauchly’s test of sphericity (W) was not significant.

## Results

**Figure 2:**
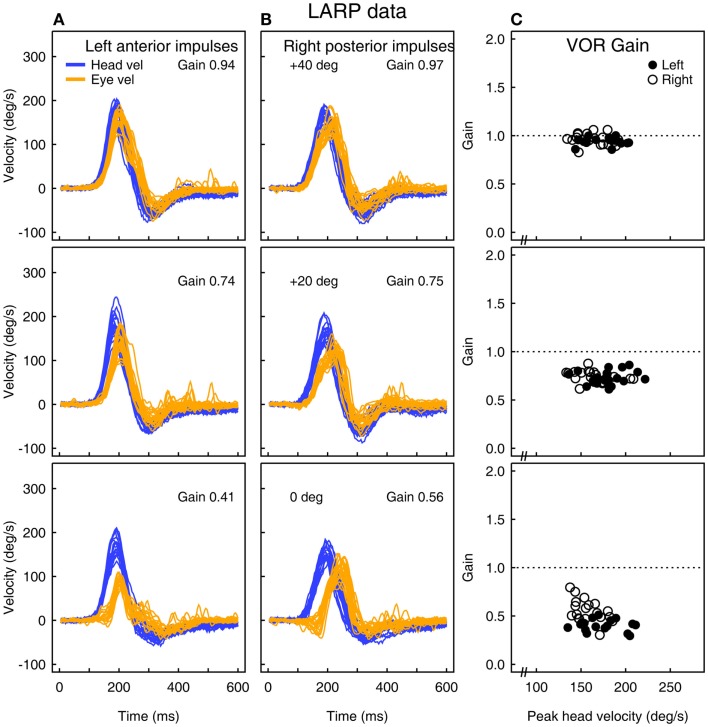
**(A,B)** Raw time series of eye and head velocity for a number of head impulses (superimposed) of one subject at three horizontal gaze angles – aligned with the canal (40°) and at 20° and 0°. The usual convention is followed – eye velocity has been inverted to show how closely it follows head velocity. The VOR gain decreases as horizontal gaze moves away from the canal LARP plane **(C)**, and at 0° it appears that the whole eye velocity response is delayed.

Figure 2 shows the head velocity and eye velocity raw data for one typical subject at three gaze positions: 40°, 20°, and 0°. As gaze is directed horizontally away from canal alignment (40°), the peak vertical eye velocity declines and so the measured LARP VOR gain decreases. However, the form of the eye velocity record changes as gaze is moved horizontally away from LARP canal plane alignment – the peak eye velocity response decreases and appears to have a delay relative to head velocity. This kind of pattern of an apparently “delayed” eye velocity response is an indication that the horizontal gaze position is not adequate. Notice also that at extreme gaze angles (e.g., 0° – straight ahead) the eye velocity is very small, and so VOR gain is very small, although the canal stimulus is just the same as for 40°. One indicator that this VOR gain measure is not valid, is that although the measured VOR gain is so small, there are no covert or overt saccades to corroborate that apparent peripheral loss of semicircular canal function. Here, the apparently reduced vertical VOR gain is a consequence of the oculomotor kinematics – the torsional component is large, so the total eye speed matches head speed, and so there is no need for any corrective saccade.

**Figure 3:**
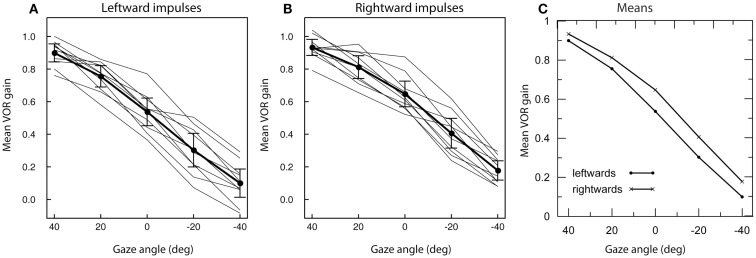
**The effect of different horizontal gaze angles on average measured vertical VOR gain for LARP impulses for 10 subjects**. Means for individual subjects are shown by the light traces; the heavy trace shows the mean across subjects, with error bars for the two-tailed 95% confidence intervals of the mean. A gaze angle of 40° corresponds to the standard LARP testing protocol, with the gaze direction aligned with the LARP canal plane; 0° corresponds to gaze straight ahead. For both leftward impulses **(A)** and rightward impulses **(B)** a clear decline of gain with increasing gaze angle is apparent **(C)**.

The results for the ANOVA are given in Table 1, and Figures 3A,B show the within-subject mean VOR gain for every subject as well as the between-subject means and two-tailed 95% confidence intervals for the mean at each gaze angle.

**Table 1:** **Experiment 1: results of analysis of variance**.

Source	Sum of squares	df	Mean square	*F*	Sig.
Gaze direction	7.950	4	1.987	234.936	0.000
Error (gaze direction)	0.305	36	0.008		
Impulse direction	0.246	1	0.146	13.874	0.005
Error (impulse direction)	0.095	9	0.011		
Gaze direction × impulse direction	0.020	4	0.005	2.879	0.036
Error (gaze direction × impulse direction)	0.064	36	0.002		

*The main effects of gaze direction and impulse direction are both significant. The interaction between gaze direction and impulse direction is borderline significant and occurs because the effect of impulse direction is slightly smaller at 40° than at other angles (see Figure 3C)*.

The main factor of gaze direction angle was significant (*F* = 234.94, df: 4.36; *p* < 0.001), as was the main factor of impulse direction (*F* = 13.874, df: 1.9; *p* < 0.005). The gaze direction × impulse direction interaction just attained significance (*F* = 2.879, df: 4.36; *p* = 0.036). Inspection of the means (Figure 3C) showed this was because at 40° the leftward and rightwards means were very similar, whereas at other gaze angles there was an almost constant difference between the two directions. At all gaze direction angles, the mean of the leftward impulses was smaller than the mean of the rightward impulses; however, at the 40° gaze angle, this difference was (slightly) reduced.

When the gaze direction is at −40°, gaze is approximately aligned with the orthogonal RALP plane, and so the vertical eye movement should have little drive from the LARP canals, but the measured vertical VOR gain is not 0. One reason may be that the canals are not exactly orthogonal, so it is virtually impossible to produce a pure LARP plane stimulus without any RALP plane stimulation. Another factor is the fact that the vertical canals have systematic curvature so they do not lie in a single plane (12).

## Discussion

With exactly the same semicircular canal stimulus, as the subject’s eye position moves away from alignment with the plane of the vertical canals being tested, the measured vertical VOR gain decreases. If gaze is straight ahead, the measured vertical VOR gain is reduced to about 0.5, which, if true, would be indicative of vestibular loss. However, it is due to oculomotor kinematics, not to decreased peripheral vestibular function, as shown by two facts: the raw data shows the same subjects have perfectly normal measured vertical VOR gain with other gaze directions, and although the VOR gain reaches the apparently low value of 0.5, there are no covert or overt saccades to corroborate that apparent peripheral loss of semicircular canal function.

### Why test vertical canals?

Information about vertical semicircular canal function allows the physician to refine the clinical diagnosis and determine whether the entire vestibular nerve is affected by neuritis, or just branches of the nerve. “Classic” superior vestibular neuritis affects the nerves from both the anterior and lateral canal. Inferior vestibular neuritis affects the nerve from the posterior canal. So involvement of both the anterior and lateral canal supports the diagnosis of “classic” superior vestibular neuritis (13). Evidence of isolated loss of posterior canal function, on the other hand, confirms the diagnosis of inferior vestibular neuritis. Vertical VOR testing evaluates superior canal function in patients with superior canal dehiscence or confirms posterior canal occlusion after such surgery for intractable benign paroxysmal positional vertigo. Combined with the new vestibular evoked myogenic potential testing (VEMP), it means that the function of all vestibular sense organs can now be tested (14).

## Conclusion

In testing vertical VOR with vHIT, the horizontal gaze direction must be aligned with the canal plane being tested. Poor gaze alignment leads to an apparently reduced VOR gain. The tell-tale signs of poor gaze alignment are

1. the appearance of an apparently “delayed” eye velocity trace;
2. the absence of any corrective saccades even when the measured VOR gain appears small.

## Author Contributions

LM, HM, and IC designed the experiment, which was carried out by LM and MM. LM, MM, AB, and IC analyzed the data and prepared figures; all authors were involved in the interpretation of the data. IC wrote the paper, and all authors revised it.

## Conflict of Interest Statement

Ian S. Curthoys, Leigh A. McGarvie, Hamish G. MacDougall, and Ann M. Burgess are currently receiving a project grant (App1046826) from NHMRC of Australia. Ian S. Curthoys is currently receiving a project grant from the Garnett Passe and Rodney Williams Memorial Foundation (Grant RP228); this grant helps to pay the salary of Ann M. Burgess. Hamish G. MacDougall is currently receiving a grant from the Garnett Passe and Rodney Williams Memorial Foundation. Ian S. Curthoys, Leigh A. McGarvie, and Hamish G. MacDougall are unpaid consultants to and have received funding for travel from GN Otometrics.

## Abbreviations

LARP, the plane of the left anterior-right posterior canals; RALP, the plane of the right anterior-left posterior canals; vHIT, video head impulse test; VOR, vestibulo-ocular reflex.

## References

[1] Curthoys IS MacDougall HG McGarvie LA Weber KP Szmulewicz D Manzari L The video head impulse test (vHIT). In: Jacobson GP Shepard NT, editors. Balance Function Assessment and Management. San Diego, CA: Plural Publishing (2014). p. 391–430.

[2] MacDougall HG McGarvie LA Halmagyi GM Curthoys IS Weber KP. Application of the video head impulse test to detect vertical semicircular canal dysfunction. Otol Neurotol (2013) 34:974–9. 10.1097/MAO.0b013e31828d676d 23714711

[3] MacDougall HG McGarvie LA Halmagyi GM Curthoys IS Weber KP. The video head impulse test (vHIT) detects vertical semicircular canal dysfunction. PLoS One (2013) 8:e61488. 10.1371/journal.pone.0061488 23630593 PMC3632590

[4] MacDougall HG Weber KP McGarvie LA Halmagyi GM Curthoys IS. The video head impulse test: diagnostic accuracy in peripheral vestibulopathy. Neurology (2009) 73:1134–41. 10.1212/WNL.0b013e3181bacf85 19805730 PMC2890997

[5] Blanks RH Curthoys IS Markham CH. Planar relationships of the semicircular canals in man. Acta Otolaryngol (1975) 80:185–96. 10.3109/00016487509121318 1101636

[6] Aw ST Halmagyi GM Haslwanter T Curthoys IS Yavor RA Todd MJ. Three-dimensional vector analysis of the human vestibuloocular reflex in response to high-acceleration head rotations. 2. Responses in subjects with unilateral vestibular loss and selective semicircular canal occlusion. J Neurophysiol (1996) 76:4021–30. 8985897 10.1152/jn.1996.76.6.4021

[7] Cremer PD Halmagyi GM Aw ST Curthoys IS McGarvie LA Todd MJ Semicircular canal plane head impulses detect absent function of individual semicircular canals. Brain (1998) 121:699–716. 10.1093/brain/121.4.699 9577395

[8] Cremer PD Migliaccio AA Pohl DV Curthoys IS Davies L Yavor RA Posterior semicircular canal nystagmus is conjugate and its axis is parallel to that of the canal. Neurology (2000) 54:2016–20. 10.1212/WNL.54.10.2016 10822450

[9] Cremer PD Minor LB Carey JP Della Santina CC. Eye movements in patients with superior canal dehiscence syndrome align with the abnormal canal. Neurology (2000) 55:1833–41. 10.1212/WNL.55.12.1833 11134382

[10] Migliaccio AA Cremer PD. The 2D modified head impulse test: a 2D technique for measuring function in all six semi-circular canals. J Vestib Res (2011) 21:227–34. 10.3233/VES-2011-0421 21846955

[11] Field A Discovering Statistics Using SPSS. 3rd ed London: Sage (2009). 856 p.

[12] Bradshaw AP Curthoys IS Todd MJ Magnussen JS Taubman DS Aw ST A mathematical model of human semicircular canal geometry: a new basis for interpreting vestibular physiology. J Assoc Res Otolaryngol (2010) 11:145–59. 10.1007/s10162-009-0195-6 19949828 PMC2862918

[13] Halmagyi GM Weber KP Curthoys IS Vestibular function after acute vestibular neuritis. Restor Neurol Neurosci (2010) 28:37–46 10.3233/RNN-2009-0533 20086281

[14] Curthoys IS. The interpretation of clinical tests of peripheral vestibular function. Laryngoscope (2012) 122:1342–52. 10.1002/lary.23258 22460150

